# Use of Triggers on in vitro Fertilization and Evaluation of Risk Factors for Sub-Optimal Maturation Rate

**DOI:** 10.1055/s-0041-1741455

**Published:** 2022-02-02

**Authors:** Larissa Matsumoto, Lucas Yugo Shiguerhara Yamakami, Edson Guimarães Lo Turco, Cristina Laguna Benetti-Pinto, Daniela Angerame Yela

**Affiliations:** 1 Department of Human Reproduction, VidaBemVinda Human Reproduction Care Center, São Paulo, SP, Brazil; 2 Department of Human Reproduction, Escola Paulista de Medicina, Universidade Federal de São Paulo, São Paulo, SP, Brazil; 3 Department of Gynecology and Obstetrics, Faculty Medical Sciences, Universidade Estadual de Campinas, Campinas, SP, Brazil

**Keywords:** dual trigger, GnRH agonist, HCG in vitro fertilization, oocyte maturation, gatilho duplo, agonista de GnRH, HCG fertilização in vitro, maturação oocitária

## Abstract

**Objective** To compare the oocyte maturation rate in the treatment of in vitro fertilization (IVF) in terms of the use of human chorionic gonadotropin (hCG), agonist gonadotropin-releasing hormone (GnRH) and dual trigger and to evaluate the associated risk factors for sub-optimal maturation rates.

**Methods** A retrospective cohort study with 856 women who underwent IVF. They performed oocyte retrieval and were classified into 3 groups (1 - hCG, 2 - GnRH agonist, 3 - dual trigger). The primary outcome was maturation rate per trigger, and the secondary outcomes were the pregnancy rate per oocyte retrieval and the correlations between low maturation rate as well as the clinical and treatment characteristics of women.

**Results** The maturation rate was 77% in group 1; 76% in group 2, and 83% in group 3 ( *p* = 0.003). Group 2 showed women with better ovarian reserve, greater number of oocytes collected, and more mature oocytes and embryos compared with the other groups ( *p* < 0.001). The cumulative clinical pregnancy rate was no different between the groups ( *p* = 0.755). Low ovarian reserve and low doses of follicle-stimulating hormone (FSH) administered during the stimulus were associated with a higher chance of null maturation rate.

**Conclusion** The oocyte maturation rates and IVF results were similar in all groups. Low ovarian reserve is associated with the worst treatment results.

## Resumo

**Objetivo** Comparar a taxa de maturação oocitária no tratamento de fertilização in vitro (FIV) em relação so o uso de gonadotrofina coriônica humana (hCG), agonista de hormônio liberador de gonadotrofina (GnRH), e gatilho duplo e avaliar os fatores de risco associados a taxas de maturação subótimas.

**Métodos** Estudo de coorte retrospectivo com 856 mulheres submetidas à FIV. Elas foram classificadas em 3 grupos (1 - hCG, 2 - GnRH agonista, 3 - gatilho duplo). O desfecho primário foi a taxa de maturação por gatilho, e os desfechos secundários foram a taxa de gravidez por recuperação de oócitos e as correlações entre a baixa taxa de maturação bem como as características clínicas e do tratamento das mulheres.

**Resultados** A taxa de maturação foi de 77% no grupo 1; 76% no grupo 2, e 83% no grupo 3 ( *p* = 0,003). O grupo 2 apresentou mulheres com melhor reserva ovariana, maior número de oócitos coletados, oócitos maduros, e embriões, em comparação aos demais grupos ( *p* < 0,001). A taxa cumulativa de gravidez clínica não foi diferente entre os grupos ( *p* = 0,755). Baixa reserva ovariana e baixas doses de hormônio folículo-estimulante (FSH) administradas durante o estímulo foram associadas a uma maior chance de taxa de maturação nula.

**Conclusão** As taxas de maturação oocitárias e os resultados de FIV foram semelhantes em todos os grupos. A baixa reserva ovariana está associada aos piores resultados do tratamento.

## Introduction

Favorable in vitro fertilization (IVF) results are related to satisfying results after ovarian stimulation, lower rates of ovarian hyperstimulation syndrome (OHSS), and lower rates of multiple pregnancies. 1 The number of mature eggs captured in in vitro fertilization is directly related to the chances of pregnancy. 2 The number of mature eggs and the risk of OHSS are directly related to the choice of the trigger. The most commonly used ovulation trigger is human chorionic gonadotropin (hCG). 3 However, the use of hCG can increase the risk of OHSS. A safer way to perform oocyte maturation is by using the gonadotropin-releasing hormone (GnRH) agonist, because it can reduce severe forms of OHSS by 50%; however, it requires intense luteal phase support. 4 5

When maturation rates are lower than 70%, there may be impairment in the outcome of IVF. 6 There is a hypothesis that a low ovarian reserve and age are related with higher chances of inadequate response to maturation trigger. 1

When the inadequate responses occur, there is the possibility of a new double trigger cycle that uses hCG and GnRH agonists. 7 The dual trigger presents better results regarding the number of mature oocytes collected and the oocyte quality, higher quantity of mature eggs, and better embryonic quality. 8

Few studies compare the three types of triggers together. Thus, the present study aims to evaluate oocyte maturation rates and IVF results according to the use of the three types of triggers to decide the choice of the trigger, offering better indication with lower risk and, thus, personalizing the treatment of each woman.

## Methods

A retrospective observational cohort study was performed with 856 women submitted to IVF at a private clinic in São Paulo, who performed the oocyte abstractions between October 2011 and July 2017.

The women were classified into three groups according to the type of trigger used: 1 - hCG (525 women); 2 - GnRH agonist (271 women) and 3 - dual trigger (60 women). A dosage of 5,000 IUs of hCG was used in group 1; the second group received a dose of 191.2 micrograms of triptorelin as the agonist GnRH; the last group, dual trigger, received hCG doses between 1,500 and 6,500 IUs in association with a dose of 191.2 micrograms of triptorelin as the agonist GnRH, with 36 hours before oocyte uptake.

The standard medication for egg maturation is hCG. However, the choice of medication to be used depends on the risk assessment of the patient developing ovarian hyperstimulation syndrome. The risk is identified through test results in which the patient has more than 25 developing follicles, estradiol above 3,500 pg/dl on the trigger day and when the anti-Mullerian hormone (AMH) dosage > 3.4 ng/ml, which leads to the use of agonist GnRH. For patients with a low ovarian reserve and advanced age, the dual trigger was chosen as an option seeking better results. The analyzed variables were age, body mass index (BMI), infertility time, follicle-stimulating hormone (FSH) and luteinizing hormone (LH) dosages (hormones dosed up to 3 days of menstruation), AMH, antral follicle count (AFC: count of total ovarian follicles of size 3–9 mm, performed by ultrasound during the early follicular phase), ovarian reserve, the number of oocytes collected, the number of metaphase II (MII) oocytes, the maturation rate (ratio between a total of MII oocytes divided by the number of total oocytes × 100), the number of zygotes, fertilization rate, and biochemical pregnancy defined by the presence of positive β-hCG and clinically defined by the presence of gestational sac on ultrasound.

Serum dosages of FSH and LH were evaluated by the Immulite System kit (Diagnostic Products Corporation, DPC. Los Angeles, CA, USA) using the chemiluminescence method and expressed in IUs/ml; the AMH serum levels were obtained by the electrochemiluminescence method, and its values were expressed in nanograms per milliliters.

The ovarian reserve was evaluated according to the criteria of Bologna 9 that defines a woman with low ovarian reserve as a woman who presents at least two of the three criteria: age greater than or equal to 40 years, low count of antral follicles or low AMH, and history of poor ovarian response in previous treatments. All data were obtained from the analysis of the medical records of these women.

The exclusion criteria were women who used the long agonist protocol as a stimulus, canceling the cycle before using the trigger, women who showed no follicular growth between 7 and 10 days of stimulation, and women whose records had up to 50% of missing data and whose spermatozoa for IVF came from testicular biopsy. We calculated cumulative pregnancy rates by the stimulus; embryonic transfers were performed fresh or with a frozen embryo, but, in both cases, we performed luteal phase support.

The study was approved by the research ethics committee of the institution under the CAAE number: 80882517.0.0000.5404. The sample size was for convenience. For the calculation of sample power, the average comparison method between 3 groups was used, considering the average and standard deviation of the maturation value (transformed in ranks due to the absence of normal distribution) in each group, and setting the significance level or type I error as 5%. Thus, we obtained a sampling power of 77.7% for the maturation rate. To compare the categorical variables between the groups, the Chi-Squared or Fisher exact tests were used. The Mann-Whitney and the Kruskal-Wallis tests were used to compare the numerical variables. Analysis of variance (ANOVA) was performed to compare the variables separated by age group. To analyze the factors related to the lower maturation rate, the Poisson regression analysis, univariate and multivariate, were used, with the stepwise criterion for selecting variables and estimating the prevalence ratio and 95% confidence interval. The significance level adopted for the statistical tests was 5%.

## Results

The frequency of triggers used in IVF was 61.33% hCG, 31.65% agonist GnRH, and 7.01% dual trigger. The mean age of the women was 35.83 ± 4.59 years, and the time of infertility considered was 36.43 ± 34.24 months. The women in the dual trigger group had older age and longer time of infertility in relation to the other groups ( *p* = 0.001 and *p* = 0.006 respectively). About the criteria used to evaluate the ovarian reserve, the HCG group had higher FSH and lower AMH values compared with the agonist GnRH group ( *p* < 0.001, *p* = 0.003 respectively) and the dual trigger group had lower AFC levels than the other two groups ( *p* < 0.001) ( Table 1 ). There was a statistical difference between the variables that indicate ovarian reserve, such as AFC ( *p* = 0.001), baseline FSH ( *p* = 0.001), and AMH levels ( *p* = 0.033) ( Table 1 ).

**Table 1:** Clinical and laboratorial characteristics of the 856 women submitted to in vitro fertilization

Variables	Total ( *n* = 856)	HCG ( *n* = 525)	Agonist GnRH ( *n* = 271)	Double trigger ( *n* = 60)	*P* *	*P* **
	*Average ± SD*	*Average ± SD*	*Average ± SD*	*Average ± SD*		
Age (years) ^b, c^	35.93 ± 4.59	36.18 ± 4.56	34.97 ± 4.57	38.12 ± 3.83	< 0.001	—
Infertility period (months) ^b^	36.43 ± 34.24	38.18 ± 35.38	32.01 ± 32.99	40.37 ± 27.55	0.006	0.016
BMI (kg/m2) ^b, c^	25.33 ± 4.84	25.36 ± 4.40	25.87 ± 5.93	22.73 ± 4.80	0.029	0.028
Basal FSH (UI/L) ^a^	8.76 ± 8.09	9.31 ± 9.22	7.84 ± 6.18	8.14 ± 3.73	< 0.001	0.002
Basal LH (UI/L)	6.55 ± 6.43	6.76 ± 7.24	6.43 ± 5.26	5.34 ± 2.73	0.369	0.388
AMH (ng/ml) ^a^	1.39 ± 1.88	1.10 ± 1.70	2.12 ± 2.45	1.29 ± 1.00	0.033	0.042
AFC (unit) ^a, b, c^	11.63 ± 7.58	10.21 ± 6.30	15.00 ± 8.91	8.63 ± 6.06	< 0.001	< 0.001

Abbreviations: AFC, count of antral follicles; AMH, anti-Mullerian hormone; BMI, body mass index; FSH, follicle stimulating hormone; LH, luteotropic hormone; SD, standard deviation.

*Kruskal-Wallis test. **Age-adjusted analysis of variance (ANOVA); **a** : difference between groups 1 and 2; **b** : difference between groups 2 and 3; **c** : difference between groups 1 and 3.

The agonist GnRH group had a greater number of total follicles (15.52 ± 8.80), a higher number of follicles larger than 16 mm (7.08 ± 4.46), greater number of oocytes collected (14.12 ± 9.37), and higher number of oocytes in MII (10.73 ± 7.39) comparing to the other groups ( *p* < 0.001), and this result remains even after separating them by age group ( Table 2 ). The maturation rate was higher with the use of the dual trigger (0.83 ± 0.17) when compared with the other groups ( *p* = 0.035), but when the groups were separated by age group, we found no difference in the rate of maturation ( *p* = 0.161) ( Table 2 ).

**Table 2:** Laboratory and characteristics of the stimulus of the three groups of triggers in women submitted to in vitro fertilization

Variables	Total ( *n* = 856)	HCG ( *n* = 525)	Agonist GnRH ( *n* = 271)	Double trigger ( *n* = 60)	*P* *	*P* **
	*Average ± SD*	*Average ± SD*	*Average ± SD*	*Average ± SD*		
Stimulus (days) ^b, c^	10.82 ± 2.01	10.62 ± 2.00	10.94 ± 1.85	12.03 ± 2.27	< 0.001	< 0.001
FSH dosage (UI) ^b, c^	2,054.30 ± 653.77	2,043.30 ± 579.43	1,936.1 ± 685.38	2,682.10 ± 768.82	< 0.001	< 0.001
LH dosage (UI) ^b, c^	941.24 ± 572.44	922.20 ± 558.96	873.89 ± 503.65	1,391.10 ± 749.88	< 0.001	< 0.001
E2 trigger (pg/ml) ^a, b^	2,249.90 ± 2,979.0	1,676.70 ± 1,334.30	3,310.30 ± 4,462.60	1,455.20 ± 944.10	< 0.001	< 0.001
P2 trigger (ng/ml) ^b, c^	2.25 ± 6.75	1.01 ± 0.5	1.10 ± 1.80	3.66 ± 1.74	< 0.001	< 0.001
LH start (UI/L)	4.99 ± 1.65	5.12 ± 2.1	4.91 ± 5.24	4.31 ± 3.78	0.300	0.196
LH trigger (UI/L)	5.33 ± 3.28	6.2 ± 4.23	4.03V6.41	4.23 ± 5.30	0.159	0.189
Total follicles ^a, b^	11.57 ± 7.23	9.71 ± 5.52	15.52 ± 8.80	9.97 ± 5.17	< 0.001	< 0.001
Follicles > 16mm ^a, b^	5.16 ± 3.67	4.22 ± 2.82	7.08 ± 4.46	4.65 ± 2.83	< 0.001	< 0.001
Oocytes collected ^a, b, c^	10.11 ± 7.82	8.46 ± 6.23	14.12 ± 9.37	6.72 ± 5.71	< 0.001	< 0.001
MII ^a, b^	7.65 ± 5.96	6.32 ± 4.50	10.73 ± 7.39	5.43 ± 4.57	< 0.001	< 0.001
Maturation rate ^b^	0.77 ± 0.22	0.77 ± 0.24	0.76 ± 0.20	0.83 ± 0.17	0.035	0.161
2PN ^a, b^	5.39 ± 4,07	4.62 ± 3.45	7.11 ± 4.69	4.57 ± 3.96	< 0.001	< 0.001
D3 Embryos ^a, b^	5.29 ± 4.17	4.50 ± 3.52	6.99 ± 4.82	4.37 ± 3.98	< 0.001	< 0.001
Blastocysts ^a, b^	2.01 ± 2.51	1.63 ± 2.08	2.80 ± 3.08	1.80 ± 2.41	< 0.001	< 0.001

Abbreviations: 2PN, zygote with two pro nuclei; D3, total embryos on the third day; E2, estradiol dosage; LH beginning, dosage of luteotropic hormone at the beginning of the stimulus; LH trigger, dosage of luteotropic hormone on the day of the trigger; MII, ova in metaphase II; P2, dosage of progesterone; SD, standard deviation. * Kruskal-Wallis test. **Age-adjusted analysis of variance (ANOVA); **a** : difference between groups 1 and 2; **b** : difference between groups 2 and 3; **c** : difference between groups 1 and 3.

The total cumulative pregnancy rate was 47.04%, 11.1% of which were twin pregnancies. There was no statistical difference between pregnancy rates ( *p* = 0.755), the number of gestational sacs ( *p* = 0.648), and the number of abortions ( *p* = 0.227) among the three groups ( Table 3 ).

**Table 3:** Results obtained after the in vitro fertilization procedure in the women of the three groups of triggers ( *n* = 856)

Variables	HCG ( *n* = 525)	Agonist GnRH ( *n* = 271)	Double trigger ( *n* = 60)	*P* *
	*n/n total*	*n/n total*	*n/n total*	
Β-HCG	211/459–46%	106/217–49%	17/34–50%	0.755
Clinical pregnancy	194/456–43%	89/213–42%	16/34–47%	0.284
Twinning	50/211–24%	26/89–29%	42,401–13%	0.227
Miscarriage	39/455–09%	27/217–12%	12,510–12%	0.272

Notes: *Chi-squared test

The null maturation rate was 3.03% and the maturation rate < 70% was 28.18%. None of the variables, such as age, infertility time, low ovarian reserve, trigger, BMI, FSH dose, LH dosed on the trigger day, or the LH dose administered in the stimulus, were significantly associated with the low maturation rate by univariate and multivariate analysis ( Table 4 ).

**Table 4:** Clinical and laboratory factors associated with the prevalence of low maturation rate ( *n* = 823)

Variables	Categories	*P* -value	PR (CI 95%) *
Ages (years)	< 30 (ref.)	—	1.00 (—)
	30–39	0.847	1.04 (0.68–1.61)
	≥ 40	0.178	0.71 (0.42–1.17)
Infertility period (months)	—	0.101	1003 (0.999–1.007)
Low ovarian reserve	No (ref.)	—	1.00 (—)
	Yes	0.786	1.04 (0.80–1.34)
Group/Trigger	Double trigger (ref.)	—	1.00 (—)
	Agonist GnRH	0.499	1.23 (0.68–2.21)
	HCG	0.202	1.44 (0.82–2.54)
BMI (Kg/m2)	< 20	0.703	0.86 (0.39–1.88)
	20.0–24.9 (ref.)	—	1.00 (—)
	25.0–29.9	0.181	1.30 (0.89–1.90)
	≥ 30.0	0.053	1.53 (0.99–2.36)
LH trigger (UI)	< 1.00 (ref.)	—	1.00 (—)
	1.00–1.99	0.905	1.03 (0.66–1.59)
	2.00–3.79	0.419	0.83 (0.52–1.31)
	≥ 3.80	0.590	0.88 (0.56–1.39)
FSH dose (UI/L)	≥ 2,475 (ref.)	—	1.00 (—)
	2,025–2,474	0.390	1.16 (0.83–1.64)
	1,650–2,024	0.794	1.05 (0.72–1.53)
	< 1,650	0.096	1.35 (0.95–1.92)
LH dose (UI/L)	< 600 (ref.)	—	1.00 (—)
	600–884	0.268	0.82 (0.57–1.17)
	885–1,349	0.829	0.96 (0.67–1.38)
	≥ 1,350	0.923	0.98 (0.70–1.38)

Abbreviations: BMI, body mass index; CI, confidence interval; FSH, follicle stimulating hormone; LH, luteinizing hormone; PR, prevalence ratio; ref, reference.

* stepwise criteria for selection of variables.

Regarding the null maturation rate when we performed the univariate analysis, the factors associated with the rate were low ovarian reserve, the LH trigger (≥ 3.80 IU), the FSH dosage (< 1,650 IUs/L) and the LH dosage (< 600 IUs/L and LH between 885–1349 IUs/L). From the results of the multivariate analysis, it was found that the low ovarian reserve and FSH dosage variables were significantly associated with the null maturation rate. The women with the highest risk of zero maturation rate were those with a low ovarian reserve (risk 4.58 times higher) and those with a low dose of FSH (< 1,650 IUs/L) (risk 16.03 times higher) ( Table 5 ).

**Table 5:** Clinical and laboratory factors associated with prevalence rate of null maturation ( *n* = 823)

Variables	Categories	*P* -value	Crude PR (CI 95%) *	Adjusted PR (CI 95%) *
Ages (years)	< 30 (ref.)	—	1.00 (—)	
	30–39	0.128	4.68 (0.29–76.86)	
	≥ 40	0.096	5.84 (0.34–100.90)	
Infertility period (months)	—	0.411	0.993 (0.978–1.009)	
Low ovarian reserve	No (ref.)	—	1.00 (—)	1.00(—)
	Yes	0.001	3.95 (1.70–9.15)	4.58 (1.77–11.90)
Group/Trigger	Double trigger (ref.)	—	1.00 (—)	
	Agonist	0.215	3.28 (0.19–56.54)	
	HCG	0.152	4.15 (0.25–67.97)	
BMI (Kg/m2)	< 20	0.294	0.43 (0.03–7.22)	
	20.0–24.9 (ref.)	—	1.00 (—)	
	25.0–29.9	0.496	0.63 (0.17–2.38)	
	≥ 30.0	0.857	1.13 (0.30–4.26)	
LH trigger (UI)	< 1.00 (ref.)	—	1.00 (—)	
	1.00–1.99	0.324	0.34 (0.0–8.31)	
	2.00–3.79	0.224	3.90 (0.44–34.87)	
	≥ 3.80	0.028	10.09 (1.29–78.81)	
FSH dose (UI/L)	≥ 2,475 (ref.)	—	1.00 (—)	1.00 (—)
	2,025–2,474	0.187	4.25 (0.50–36.33)	5.78 (0.67–49.85)
	1,650–2,024	0.177	4.52 (0.51–40.46)	6.36 (0.71–57.25)
	< 1,650	0.008	15.83 (2.08–120.35)	16.03 (2.07–124.32)
LH dose (UI/L)	< 600	0.049	4.74 (1.01–22.30)	
	600–884	0.257	2.58 (0.50–13.31)	
	885–1,349	0.031	5.39 (1.16–24.92)	
	≥ 1,350 (ref.)	—	1.00 (—)	

Abbreviations: BMI, body mass index; CI, confidence interval; FSH, follicle stimulating hormone; LH, luteinizing hormone; PR, prevalence ratio; ref, reference.* stepwise criteria for selection of variables.

## Discussion

We observed heterogeneity among the groups: the women who used the dual trigger were older and with a longer time of infertility, and the group that used agonist GnRH had a better ovarian reserve (higher AMH and AFC values and lower values of base FSH).

Up to 37% of IVF treatments are performed in patients with advanced age. It was known that age is a prognostic factor in the results of IVF. Studies show that with increasing age there are worse results in the treatment of IVF. 10

We separate the results by age group to avoid this bias. However, the agonist GnRH group had a greater number of total follicles, a greater number of follicles with a size larger than 16 mm, a greater number of oocytes, and a higher number of MII oocytes than the other groups. This can be explained because women in this group have the highest ovarian reserves.

The choice of the trigger is of the responsibility of the physician who conducts the treatment, and who obeys a hyperstimulation prevention protocol, in which the use of agonist GnRH and freeze all strategy are safe conducts. 11 This justifies the choice of the use of agonist GnRH for younger women those with a better ovarian reserve, as they are the ones that present a higher risk of hyperstimulation syndrome. Thus, it was expected that those were also the women with better results in the absolute number of oocytes collected and MII oocytes, as observed.

This group also showed a greater number of fertilized oocytes, and 3 ^rd^ - and 5 ^th^ -day embryos compared with the other two groups, which explains the use of agonist GnRH alone for oocyte maturation—as oocyte quality would not change—affecting the outcome of IVF, as shown by some. 12

Despite the group 2 results, the dual trigger group has a higher oocyte maturation rate, though, when adjusted by age groups, we found that the three groups had similar rates of maturation.

In the other study, the randomized clinical trial showed a higher maturation rate with the agonist GnRH use over hCG use. 11 Another study comparing the dual trigger group with the hCG group found no difference in the maturation rates between them. 13

The use of agonist GnRH is more physiologic but has a luteolytic effect, reducing the half-life of the corpus luteum, requiring more robust luteal phase support to lower implantation rates, and causing a higher risk of miscarriage in cycles transferred to fresh. 14 In our study, we did not observe a difference in the number of abortions when we compare the three groups. We also found no difference in the rate of pregnancy and twins.

A meta-analysis with 859 women shows the same pregnancy rates where fresh transfer in women who have made the use of GnRH agonist (33%) or hCG (34%). This study also found no difference in abortion rates between the groups (agonist GnRH 20%, hCG 12.5%, *p* = 0.06). 15

In our study, we found no difference in the results of IVF treatment despite the women in the dual trigger group being older and having a worse ovarian reserve.

A retrospective study comparing the dual trigger with the hCG groups in women with low ovarian reserve showed a better maturation rate with the use of the dual trigger, but found no difference in the fertilization rates, embryonic quality, and pregnancy or abortion rates. 4

Other studies show that the dual trigger has a higher maturation rate, implantation, and pregnancy rates with hCG. 6 16 In addition to the results of IVF, we sought to identify the factors associated with the worse rates of oocyte maturation to improve the number of oocytes collected, and to help guide the expectations for treatment. According to our findings the amount of mature zygotes in treatment was directly related to the success of the IVF treatment. When we observe null maturation rates, it is usually an indicator of low ovarian reserve that was evaluated by AFC, AMH, and FSH.

A lower AFC (< 11) was related to an increased risk of cycle cancellation due to low response. A study shows 12 as a cutoff point for AFC, for low response to treatment. 17 In addition to AFC, the literature discusses the role of BMI in response to treatment; overweight and obesity were associated with a poor ovarian response, with less than three oocytes retrieved. 1 However, in our study, we found no association between BMI and worse results regarding the oocyte maturation rate.

Concerning the ovarian stimulus, lower doses of FSH administered were related to a smaller number of oocytes. 12 It is theorized that the correlation of the ratio of AMH with AFC when high, reduces ovarian sensitivity response to exogenous FSH administered, and the analysis that for women with low ovarian reserve, increasing the dose of FSH administered does not improve the ovarian response, nor the pregnancy rates. 18

However, a multicenter study by Melo et al. with 1,515 patients, evaluating the cost-effectiveness of individual doses of FSH, concluded that individualization of the dose of FSH does not change the outcome of IVF, and is a more expensive treatment. 17

The use of FSH at lower doses, less than 1,650 IUs per stimulus, was one of the factors correlated with the null maturation rate. Studies with mild stimulation show a greater number of cancellations cycles than those with traditional stimulus while affording the same pregnancy rates of traditional stimulus, lead to fewer oocytes, and may present a greater risk of not finding oocytes in the oocyte retrieval in a frequency of up to 9.4%. 7 10

The group of women with worse prognosis who followed protocols with lower doses of medication might have compromised results, with lower doses of FSH during the ovarian stimulus. Despite the differences between the groups, we had a low prevalence of zero maturation rate and the IVF results were similar between of them.

The limitations of the study are due to being a retrospective analysis, and the sample evaluated not being homogeneous. Thus, the different number of women in each group and the differences in their characteristics (such as age, infertility time, BMI, and ovarian reserve) are limiting factors for the analysis. It is noted that women who used hCG can be classified as normal responders, those who chose to use agonist GnRH presented a higher risk of OHSS and those of the dual trigger group were women with low ovarian response.

## Conclusion

Despite the differences between the three evaluated groups, the rates of oocyte maturation and the IVF results were similar across the sample; this demonstrates that the adequate choice in the type of trigger used will yield good results in IVF treatments. Low ovarian reserve jeopardizes the results of IVF, increasing the chances of null maturation rates. Moreover, even on the low ovarian reserve group, the choice of the trigger is paramount to achieving greater maturation and cumulative β-hCG rates.
